# Antihyperglycemic and Antiobesity Effects of JAL2 on* db/db* Mice

**DOI:** 10.1155/2016/6828514

**Published:** 2016-03-16

**Authors:** In-Seung Lee, Ki-Suk Kim, Kang-Hoon Kim, Jiyoung Park, Hyeon-soo Jeong, Yumi Kim, Yun-Cheol Na, Won Seok Chung, Kwang-Seok Ahn, Seok-Geun Lee, Jae Young Um, Jun Hee Lee, Hyeung-Jin Jang

**Affiliations:** ^1^College of Korean Medicine, Kyung Hee University, 1 Hoegi-dong, Dongdaemun-gu, Seoul 130-701, Republic of Korea; ^2^Western Seoul Center, Korea Basic Science Institute, 150 Bugahyeon-ro, Seodaemun-gu, Seoul 120-140, Republic of Korea

## Abstract

*Lonicera japonica* Thunb. (LJT) and* Rehmannia glutinosa* Libosch. (RGL) have been used traditionally as a herbal medicine in Korean medicine. Using LC/Q-TOF was performed to profile the two herbal medicines and the mixture of LJR and RGL (JAL2, ratio 1 : 1). We performed oral glucose tolerance test (OGTT) and plasma GLP-1 and insulin secretion by multiplex assays to investigate antidiabetic effects of LJT, RGL, and JAL2 in* db/db* mice, the mice model of type 2 diabetes mellitus (T2DM). Also, the antiobesity-related factors such as plasma peptide YY (PYY), triglyceride, total cholesterol, HDL, LDL, and weight of liver, epididymal, and retroperitoneal fat tissue were investigated. Through the multiplex assay, it was found that JAL2 treatment more efficiently attenuated high levels of blood glucose by stimulating GLP-1 secretion and reduced LDL concentration and weight of liver and retroperitoneal fat tissue compared to LJT or RGL treated separately. These results suggest that the JAL2 has antidiabetes and antiobesity effects in T2DM mice model.

## 1. Introduction

Obesity, characterized by excess accumulated body fat, may result in a negative effect on health and lead to reduction in life expectancy [1]. Currently, the number of people afflicted with obesity has increased due to nutritionally rich diets, physical inactivity, and genetic susceptibility [2]. Obesity is associated with lots of diseases, that is, type 2 diabetes mellitus (T2DM), cardiovascular disease, obstructive sleep apnea, certain types of cancer, osteoarthritis, and asthma [1, 3]. Particularly, T2DM may be the most serious among them [4, 5].

The symptoms of high blood glucose level include frequent urination, increased thirst, and increased hunger; diabetes has become one of the principle causes of morbidity and mortality [6]. T2DM, the far more common type of diabetes, is the state of insulin resistance whose cells fail to respond to insulin properly [6, 7].

Insulin resistance and common property such as hypertension, hyperlipidemia, and abdominal obesity are common phenomenon in T2DM and obesity [8]. The prevalence rate of people who have both T2DM and obesity has rapidly increased all over the world every year, and the rates of increase show no signs of slowing [1, 2, 9]. Even though T2DM and obesity are a risk factor for cardiovascular disease, most of the treatment of metabolic syndrome only focuses on the reduction of blood glucose levels or the regulation of body weight.

Glucagon-like peptide-1 (GLP-1) and peptide YY (PYY) are gastrointestinal (GI) hormone that both are secreted from enteroendocrine L cells [10, 11]. GLP-1 has been thoroughly studied as a potential attenuating hyperglycemic agent because its functions induce glucose-dependent insulin secretion from pancreatic *β* cells and insulin sensitivity levels [10, 12]. PYY acts to regulate appetite by inhibiting gastric motility and raising the water absorption in the colon [11, 13]. Retarding gastric emptying and acting as satiety factors of both GLP-1 and PYY have physiologically significant functions of relevance to obesity and T2DM.

*Lonicera japonica* Thunb. (LJT) and* Rehmannia glutinosa* Libosch. (RGL) occupy the highest proportion of the nine herbal medicines that make up the Yangkyuksanhwa-tang. Yangkyuksanhwa-tang, prescribed to patients with diabetes in Korea, consists of nine herbal medicines: LJT, RGL, Forsythiae Fructus, Gardeniae Fructus, Menthae Herba, Anemarrhena Rhizome, Gypsum Fibrosum, Schizonepetae Herba, and Ledebouriellae Radix [14]. Methanol extract of* Lonicera japonica* flower has been investigated for having suppression effect of body weight gain and body fat increase [15]. Aqueous extract of RGL has been closely studied for stimulating the expression of proinsulin gene in T2DM rats [16].

In this study, we investigated the mixture of LJT and RGL (JAL2, 1 : 1) and whether 30% ethanol extracts of JAL2 attenuates hyperglycemia and reduces the body weight via stimulating GI hormones such as GLP-1 and PYY, respectively, using* db/db* mice, which has the characteristics of T2DM. This study provided the important information of the JAL2 effects to enhance GLP-1 and PYY secretion and the possibility that the herbal medicine mixture may be used as a therapeutic agent of T2DM and obesity.

## 2. Materials and Methods

### 2.1. Preparation of 30% Ethanol Extract of LJT, RGL, and JAL2

LJT, RGL, and both herbal medicine mixture were purchased from Hamsoapharm (Hamsoa Pharmaceutical Co., Ltd., Seoul, South Korea). The herb was cut down in a proper size and extracted as follows: each medicinal herbal medicine and the mixture were performed using reflux extraction with 30% ethanol (30% EtOH) for 3 h at 75°C. Filtration and evaporation were then conducted with a rotary vacuum evaporator (N-N series, EYELA, Japan) at 55°C. The solution was freeze-dried for 24 h at −80°C and lyophilized.

### 2.2. Analysis of LJT, RGL, and JAL2 Extracts by Using LC/Q-TOF

LC/Q-TOF was conducted at the Korea Basic Science Institute (KBSL, Seoul, Korea). Chromatographic separation of the two herbal medicines and JAL2 by Agilent 1290/6550 (Agilent Technologies, Waldron) was performed using a Waters BEH C_18_ column (2.1 mm × 150 mm ID, 1.7 *μ*m, Agilent). The mobile phase consisted of solvent A (water) and solvent B (acetonitrile), both containing 0.1% formic acid. The flow rate of the mobile phase was 300 *μ*L/min and the gradient program was as follows: 0–15 min (0–40% B), 15–20 min (40–95% B), and 20–27 min (95–0% B), after which the column was equilibrated with 0% B for 5 min. Samples of 1 *μ*L were injected into the column using an autosampler. The HPLC system was interfaced to the MS system, an Agilent 6550 Accurate-Mass Q-TOF (Agilent Technologies, Santa Clara) equipped with a Dual AJS ESI source operating in positive ion mode. Mass spectra were acquired at a scan rate of 1 spectrum/s with a mass range of 50 to 1,000* m/z*.

### 2.3. Animal

The* db/db* mice were used as a murine model exhibiting hyperglycemia and overweight which are typical phenotypes of T2DM and obesity. Seven-week-old male* db/db* mice with a C57BL/6 background were obtained from the Daehan Bio Link (Daehan Bio Link Co., Ltd., Eumseong-gun, Chungcheongbuk-do, South Korea). All animal studies were performed according to protocols approved by the Institutional Animal Care and Use Committee (IACUC) of Kyung Hee University (confirmation number: KHUASP(SE)-14-046). The animals were housed in the animal room where the condition of a 12 h light-dark cycle at moderate temperature (21–23°C) and humidity levels (55–60%) are maintained. All mice were acclimated for one week before the experiment. During the acclimation period, all mice were fed with the standard rodent chow (28.507% of protein, 13,496% of fat, and 57.996% of carbohydrates (LabDiet, St. Louis, MO)) and water* ad libitum*. After acclimation period, eight-week-old mice (*n* = 5 per each group) were divided into five groups: control (saline treatment), 280 mg/kg/day of metformin treatment, 30 mg/kg/day of LJT treatment, 30 mg/kg/day of RGL treatment, and 30 mg/kg/day JAL2 treatment group.

### 2.4. Oral Glucose Tolerance Test (OGTT)

At the seventh week of the experiment, all mice were fasted for 16 h before the OGTT. Each group of mice was orally administrated saline, metformin, LJT, RGL, or JAL2, after which 5 g/kg of glucose was orally administrated. Using an Accu-Chek Performa device (Roche Diagnostics, Mannheim, Germany), the blood glucose levels were measured from the tail vein at 6 time points: before the glucose gavage (time point 0 min), 10 min after the glucose gavage (time point 10 min), 20, 40, 90, and 120 min.

### 2.5. Mouse Metabolic Hormones Multiplex Assay

After OGTT, all mice were allowed to rest for four days to prevent hypotensive or hemorrhage shock. After resting phase, all mice were fasted for 16 h before the blood sampling. Each mouse group was orally administrated saline, metformin, LJT, RGL, or JAL2 just before the glucose gavage (2 g/kg). The blood samples were collected from the tail vein of each mouse at 5 time points (before the glucose time point: 0 min, 10 min after the glucose gavage: 10 min, 20, 30, and 40 min) and were transferred to an EDTA-coated 1.5 mL microcentrifuge tube containing a dipeptidyl peptidase- (DPP-) IV inhibitor (EMD Millipore Co., Billerica, MA) and a protease inhibitor cocktail (Roche Diagnostics) to protect against GLP-1 degradation and blood coagulation, respectively. Collected blood samples were centrifuged at 1,000 ×g for 10 min at 4°C. The plasma samples were carefully transferred to the fresh tube. The Milliplex Map Kit Mouse Metabolic Hormones Magnetic Bead Panel: GLP-1, insulin, and PYY, (EMD Millipore Co., Billerica, MA) was performed as described in the manufacturer's guidelines. The experimental plates were run using the MAGPIX instrument (Bio-Rad, Hercules, CA) and data were extracted with Luminex xPONENT software. Using Bio-Plex Manager software (Bio-Rad), preliminary data was checked and analyzed.

### 2.6. Measurements of Plasma Triglycerides and Cholesterol Levels

After eight weeks of treatment, whole blood of each mouse was collected via the retroorbital plexus to BD Vacutainer® Plus Plastic K_2_ EDTA tubes (BD Biosciences, San Jose, CA) and centrifuged 1,000 ×g for 20 min at 4°C. The supernatant was transferred to a fresh tube for determination. Plasma TGs, TC, LDL, and HDL levels were analyzed at the Seoul Medical Science Institute (SCL, Seoul, South Korea).

### 2.7. Measurements of Liver, Retroperitoneal Fat, and Epididymal Fat Tissue Weights

On the day of the experiment, all groups of mice were sacrificed and the livers and epididymal fat and retroperitoneal fat tissues of each mouse were enucleated and weighed.

### 2.8. Statistical Analysis

All data are represented as mean ± SEM. IBM SPSS Statistics 22 software (IBM Corporation, Armonk, NY) and Graphpad Prism 5 software (Graphpad Software, San Diego, CA) were used for statistical analysis and graphics. The statistical significance of area under curve (AUC), blood profiling, and weight of tissues graphs was measured by Mann-Whitney *U* test (one-tailed). The generalized estimating equation for repeated-measures was used to analyze the blood glucose levels and GI hormones levels to detect group-by-time interactions. Using the Mann-Whitney *U* test was done for the intergroup comparison of OGTT and plasma hormones at each time point. A group-by-time interaction was found by the generalized estimating equation (GEE). 

## 3. Results

### 3.1. LC/Q-TOF Analysis of 30% EtOH Fraction of LJT, RGL, and JAL2

**Figure 1:**
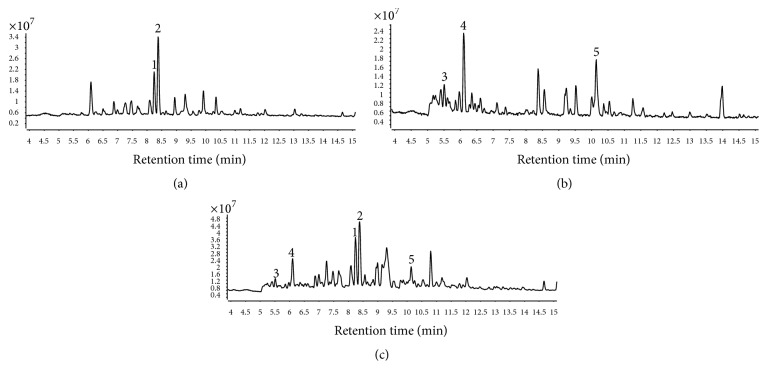
LC/Q-TOF profiling of LJT, RGL, and JAL2. To profile LJT, RGL, and the mixture of LJT and RGL (JAL2), LC/Q-TOF was performed. (a) Sweroside (1) and loganin (2) were identified in the LJT extract. (b) In the RGL extract, rehmannioside D (3), leonuride (4), and rehmaionoside A/B (5). (c) All aforementioned components were identified in JAL2.

LC/Q-TOF was performed to profile the two herbal medicines and herbal medicines mixture (Figure 1). Among the several prominent peaks, sweroside and loganin were identified as the highest peak in the LJT extract (Figure 1(a)). In the RGL extract, rehmannioside D, leonuride, and rehmaionoside A/B were identified (Figure 1(b)). All compounds mentioned above were determined in the JAL2 extract (Figure 1(c)).  Table 1 contains more detailed information about JAL2 including the LJT and RGL extract metabolites.

### 3.2. Hyperglycemia Attenuating Effects of LJT, RGL, and JAL2 on* db/db* Mice

**Figure 2:**
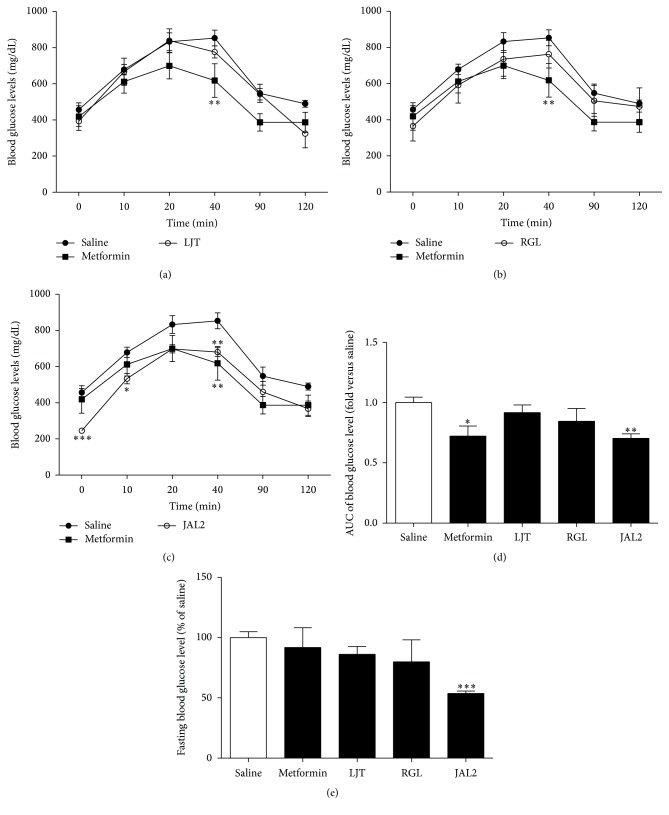
Effects of LJT, RGL, and JAL2 on blood glucose levels change and fasting blood glucose level. To test regulatory effect of LJT (a), RGL (b), and JAL2 (c), LJT, RGL, and JAL2 were orally administrated just before glucose gavage (5 g/kg) to the* db/db* mice. Metformin used as a positive control. (d) The variation of blood glucose levels of each group is indicated by a bar graph. (e) Fasting blood glucose level of each group is indicated by a bar graph. ^*∗*^ *P* < 0.05; ^*∗∗*^ *P* < 0.01; ^*∗∗∗*^ *P* < 0.001, compared with saline-treated group by Mann-Whitney *U* test. Results are represented as means ± SEM. *n* = 5 for each group.

The hyperglycemic attenuating effects of LJT, RGL, or JAL2 were investigated using the T2DM rodent model,* db/db* (Figure 2). A group-by-time interaction was noted. Blood glucose levels in most groups were peaked at 20 min from the glucose gavage process. The blood glucose levels of the LJT-treated mice showed variation tendencies similar to those of the saline-treated mice (Figure 2(a)). The RGL-treated group, compared to the saline-treated group, showed maintaining lower blood glucose levels at all times without significance (Figure 2(b)). Mice treated with JAL2 were found to exert the highest hypoglycemic effect compared to the saline-treated mice (Figure 2(c)). Metformin, known to lower blood glucose levels, was also shown to compare the efficiency with those of LJT, RGL, and JAL2 (Figures 2(a)–2(c)). AUC for representing the blood glucose levels changes during OGTT was presented to make a comparison between the groups treated with saline, met, LJT, RGL, or JAL2 (Figure 2(d)). There was a trend toward decreased fasting blood glucose levels by LJT and RGL. In the JAL2-treated group, the mixture of LJT and RGL, the fasting blood glucose levels significantly decreased compared to the saline-treated group (Figure 2(e)).

### 3.3. Regulation Effects of LJT, RGL, and JAL2 on the GI Hormones Secretion

**Figure 3:**
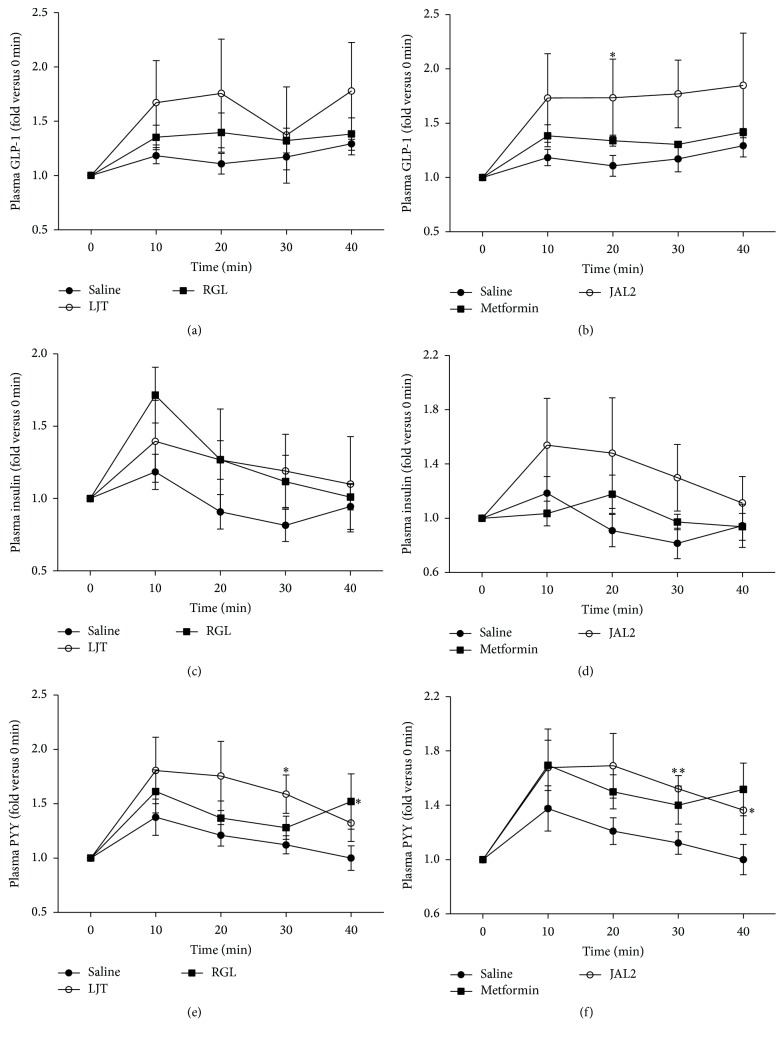
Plasma GI hormones change by LJT, RGL, and JAL2 administration. To test the stimuli effect of LJT, RGL or JAL2 was given to* db/db* mice. Before glucose administration (2 g/kg), LJT, RGL, or JAL2 was orally administrated. (a-b) Plasma GLP-1 secretion variation levels after glucose gavage. (c-d) Plasma insulin secretion variation levels after glucose gavage. (e-f) Plasma PYY secretion variation levels after glucose gavage. Metformin was used for positive control. ^*∗*^ *P* < 0.05; ^*∗∗*^ *P* < 0.01, compared with saline-treated group by Mann-Whitney *U* test. Results are represented as means ± SEM. *n* = 5 for each group.

To investigate the regulation effects of LJT, RGL, and JAL2 on the GI hormones secretion, GLP-1, insulin, and PYY, plasma was separated from the collected blood samples obtained at each time point (0, 10, 20, 30, and 40) (Figure 3). For plasma GLP-1, insulin, and PYY, a group-by-time interaction was found by the GEE. Plasma GLP-1 concentration levels were increased in all herbal medicines and met treated mice compared to the saline-treated mice: specifically, the mice are treated with JAL2 or met significantly more secreted GLP-1 20 min after glucose gavage process (2 g/kg) (Figure 3(b)). Therefore, insulinotropic action may be led by the stimulated GLP-1. Insulin secretion was increased by all herbal medicines at all times, but metformin-treated mice less secreted insulin compared to the saline-treated mice 10 min after glucose gavage process (Figure 3(d)). PYY secretion was also increased by all herbal medicines and metformin compared to the saline-treated mice. Interestingly, each LJT- or RGL-treated mouse demonstrated significant increased PYY secretion at 30 and 40 min, respectively, after glucose gavage process (Figure 3(e)). The concentration of PYY was significantly increased both 30 and 40 min after glucose gavage process in the JAL2-treated mice including LJT and RGL (Figure 3(f)).

### 3.4. Effects of LJT, RGL, and JAL2 on Blood Profiles in* db/db* Mice

**Figure 4:**
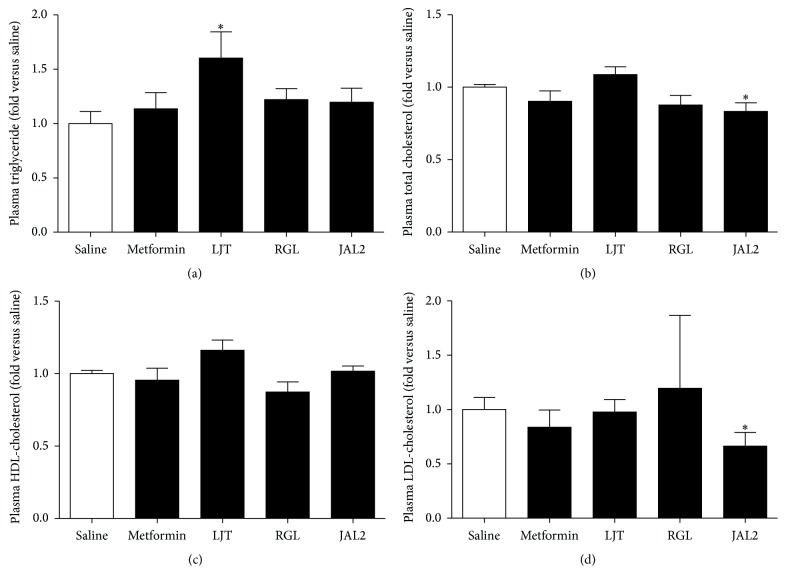
Plasma concentration of TGs, TC, HDL, and LDL in response to LJT, RGL, and JAL2. To study antiobesity effects of LJT, RGL, and JAL2 on obesity-related factors: TGs (a), TC (b), HDL (c), and LDL (d). ^*∗*^ *P* < 0.05 compared with saline-treated group by Mann-Whitney *U* test. Results are represented as means ± SEM. *n* = 5 for each group.

At the end of the treatment, whole blood of each mouse was collected from which plasma was isolated for investigating the effects of the LJT, RGL, and JAL2 on the obesity-related blood factors: plasma triglyceride (TG), total cholesterol (TC), low-density lipoprotein cholesterol (LDL-c), and high-density lipoprotein cholesterol (HDL-c) levels. Each LJT or RGL treatment did not result in significantly reducing the obesity associated factors, while the JAL2 significantly reduced TC and LDL-c levels (Figure 4).

### 3.5. Effects of LJR, RGL, and JAL2 on Liver and Fat Tissues Weight

**Figure 5:**
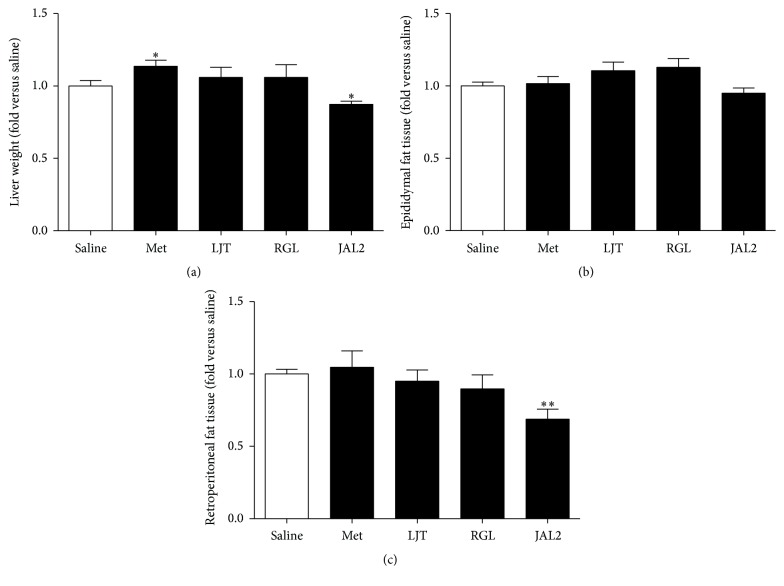
Weight of liver, epididymal, and retroperitoneal fat tissue of each experimental group. Another obesity-related factors, weight of liver (a), epididymal fat (b), and retroperitoneal fat tissue (c), of each group were measured. The significance of each experimental group was compared to the saline-treated group by Mann-Whitney *U* test. ^*∗*^ *P* < 0.05; ^*∗∗*^ *P* < 0.01. Results are represented as means ± SEM. *n* = 5 for each group.

To investigate the effects of LJT, RGL, and JAL2 on the liver and fat tissues weights, each mouse was sacrificed, after which the liver and fat tissues of each mouse were collected and weighed just after blood collection (Figure 5). Single-treatment of the LJT or RGL did not result in significantly reducing the weights of the liver and fat tissues. However, the weights of the liver and retroperitoneal fat tissues were significantly decreased by mice with JAL2 treatment.

## 4. Discussion

Abdominal obesity, raised fasting plasma glucose, and high TG levels are medical conditions followed by metabolic syndrome, related to the risk of developing cardiovascular disease (CVD) and diabetes [17–19]. Constantly, many researchers have studied to reduce the risk of metabolic disorder such as attenuating high blood glucose levels or reducing body mass index (BMI). Due to the side effects of the drugs, the purpose of a single target disease like antiobesity or antidiabetes has been investigated [20]. For example, diguanides, thiazolidinedione, and alpha glucosidase inhibitors, used for antidiabetic agents, result in renal failure, hypoglycemia, liver toxicity, abdominal distention, and abnormal weight gain. On the other hand, antiobesity agent, sibutramine, causes the cardiac vascular disease [21–23].

Therefore, oriental medicine has begun to attract the attention because of its excellent efficacy and safety compared to current drugs [24]. In this study, we investigated how the LJT, RGL, and JAL2 affected obesity and T2DM related factors such as body weight, body compositions, blood glucose level changes, and GI hormones on* db/db* mice, which have both phenotypes of obesity and T2DM [25]. Mice treated with JAL2 showed less increase changes of blood glucose levels and significant reduction of fasting blood glucose level compared to the saline-treated mice and metformin-treated mice, known to be a T2DM agent (Figures 2(c) and 2(e)). Metformin treatment did not affect fasting blood glucose levels compared to saline-treated* db/db* mice, which may be associated with an improvement in glycocalyx barrier properties [26].

In recent year, there is new trend of drugs that excite to release GLP-1 secretion or inhibit the GLP-1 degradation. GLP-1, a GI hormone, regulates the insulin secretion, appetite, and gut motility. Also, the insulin sensitivity of pancreatic beta-cells is enhanced by GLP-1 [27–29]. GLP-1 secretion in mice treatment with JAL2 was significantly increased (Figure 3(b)). Although the significance was not determined, a trend toward higher levels of plasma GLP-1 secretion during the feeding periods of LJT and RGL was determined. The two herbal medicines and JAL2 led to increase of GLP-1 secretion in* db/db* mice, thereby stimulating the insulin secretion (Figures 3(b) and 3(d)) [10]. The maintained lower fasting blood glucose levels and significantly stimulated GLP-1 secretion may prove the improvement in insulin resistance capabilities of JAL2 treatment in* db/db* mice. Another GI hormone, PYY, known to be a pancreatic peptide YY_3-36_, plays a role in decreasing appetite through inhibiting gastric motility [11, 30]. The two herbal medicines and JAL2 increased PYY secretion and had significance (Figure 3). The results of this study demonstrate the possibility of the JAL2 as a weight controller via reducing appetite.

TG, relevant to the accumulation of lipid repository in the liver, is concerned with metabolic syndrome and T2DM [31]. However, plasma TG levels were significantly increased in LJT-treated* db/db* mice (Figure 4(a)). A study reported that pioglitazone, an antidiabetic agent, successfully restored the insulin secretory capacity of pancreatic *β*-cells of obese* db/db* mice, but TG levels were not affected when compared to nontreated group [32]. Also, there was a report that metformin showed a side effect by increasing TG levels in T2DM patients [33]. It demonstrates that diabetes improving effect may be largely associated with TG levels. Lower total and LDL-cholesterol levels were found in the JAL2-treated group compared to saline-treated group (Figures 4(b) and 4(d)). The results indicate an improvement in lipid accumulation in* db/db* mice by JAL2.

Obesity, a medical condition in the excessive accumulation of body fat, is designated by BMI and total cardiovascular risk factors [34]. We investigated the effects of LJT, RGL, and JAL2 by weighing the liver, retroperitoneal fat, and epididymal fat tissues (Figure 5). Function of the liver is essential for maintaining blood glucose levels by supplying glucose as an energy source to the organs [35]. However, the rise of obesity or diabetes causes nonalcoholic fatty liver disease (NAFLD) which is a condition that causes serious liver complications [36]. For the liver, the weight of the liver tissue was significantly reduced in JAL2-treated mice (Figure 5(a)). Also, in retroperitoneal fat tissue, JAL2 significantly decreased the weight of retroperitoneal fat tissue compared to saline-treated group (Figure 5(c)). Epididymal fat, considered as a key marker of the alteration of white adipose tissue, is one of several adipose depots that compose the visceral fat, places inside the abdominal cavity [37–39]. The significance was not determined; only mice treated with JAL2 demonstrated decreasing trends toward the weights of the epididymal fat tissue (Figure 5(b)).

In conclusion, most of the drugs that aim to treat metabolic syndrome only improve one symptom such as antidiabetes or antiobesity. In this study, diabetic and obesity-related factors studies proved that JAL2 (the mixture of LJT and RGL) possessed a synergistic effect of LJT and RGL, which showed significantly improved glucose tolerance by stimulating GLP-1 secretion and inhibited the undue accumulation of body fat. Although the exact mechanism of synergy between the two herbs still requires further studies, our results recommend that JAL2 might be helpful in attenuating high blood glucose levels and reducing accumulated body fat in patients with obese-T2DM and prediabetic patients.

## Figures and Tables

**Table 1:** Characterization of LJT, RGL, and JAL2 compounds by using LC/Q-TOF.

Peak number	Compound	Formula	RT	Mass	(M + H)^+^ *m*/*z*	Sample
1	Sweroside	C_16_H_22_O_9_	8.389	358.1264	359.1337	LJT/JAL2
2	Loganin	C_17_H_26_O_10_	8.243	390.1526	391.1599	LJT/JAL2
3	Rehmannioside D	C_27_H_42_O_20_	5.511	686.2342	687.2342	RGL/JAL2
4	Leonuride	C_15_H_24_O_9_	6.107	348.142	349.1493	RGL/JAL2
5	Rehmaionoside A/B	C_19_H_34_O_8_	10.143	390.2254	391.2326	RGL/JAL2
